# Search Query Data to Monitor Interest in Behavior Change: Application for Public Health

**DOI:** 10.1371/journal.pone.0048158

**Published:** 2012-10-23

**Authors:** Lucas J. Carr, Shira I. Dunsiger

**Affiliations:** 1 Department of Health and Human Physiology, University of Iowa, Iowa City, Iowa, United States of America; 2 Centers for Behavioral and Preventive Medicine, The Miriam Hospital, Providence, Rhode Island, United States of America; Rollins School of Public Health, Emory University, United States of America

## Abstract

There is a need for effective interventions and policies that target the leading preventable causes of death in the U.S. (e.g., smoking, overweight/obesity, physical inactivity). Such efforts could be aided by the use of publicly available, real-time search query data that illustrate times and locations of high and low public interest in behaviors related to preventable causes of death.

**Objectives:**

This study explored patterns of search query activity for the terms ‘weight’, ‘diet’, ‘fitness’, and ‘smoking’ using Google Insights for Search.

**Methods:**

Search activity for ‘weight’, ‘diet’, ‘fitness’, and ‘smoking’ conducted within the United States via Google between January 4^th^, 2004 (first date data was available) and November 28^th^, 2011 (date of data download and analysis) were analyzed. Using a generalized linear model, we explored the effects of time (month) on mean relative search volume for all four terms.

**Results:**

Models suggest a significant effect of month on mean search volume for all four terms. Search activity for all four terms was highest in January with observable declines throughout the remainder of the year.

**Conclusions:**

These findings demonstrate discernable temporal patterns of search activity for four areas of behavior change. These findings could be used to inform the timing, location and messaging of interventions, campaigns and policies targeting these behaviors.

## Introduction

The three leading preventable causes of death in the U.S. are smoking, overweight/obesity and physical inactivity [1], [2]. Smoking accounts for an estimated 443,000 deaths annually followed by overweight/obesity (216,000 deaths) and physical inactivity (191,000 deaths) [2]. Collectively, these behaviors are responsible for one-third (33.3%) of all deaths in the U.S [1], [2]. The Centers for Disease Control and Prevention (CDC) have identified tobacco, physical inactivity and overweight/obesity as “Winnable Battles” for public health [3].

However, given the high prevalence rates of these behaviors, there is a need for effective interventions and policies targeting long-term maintenance of healthy behaviors. Such efforts could be aided by the use of publicly available search query data that provides insight into regional and seasonal interest in pertinent search terms related to such behaviors.

Through a free and publicly available extension known as Google Insights for Search [4], search query data for searches conducted via Google post-2004 can be analyzed both geographically and temporally. Such data could assist researchers, practitioners and policy makers in choosing the specific timing, location and messages to be used in behavioral interventions, public health campaigns and policies targeting populations in need.

Search query data has previously been demonstrated as effective for detecting influenza epidemics [5], [6], seasonal trends of depression [7] and trends in smoking habits [8], [9]. However, to our knowledge, no studies have investigated its use for exploring patterns of public interest in the areas of diet/weight and/or physical activity/fitness. The purpose of this study is to assess patterns of public interest in major behavior change topics of ‘weight’, ‘diet’, ‘fitness’, and ‘smoking’ using publicly available search query data.

## Methods

Search query data was provided by Google Insights for Search [4] and the methodology used by Google to aggregate search query data has been described previously [6]. Briefly, Google aggregates historical logs of search queries for chosen terms submitted within a chosen time frame and region. Search queries for pertinent terms are provided in a relative format (e.g., count of searches for chosen term divided by total number of searches within the chosen time frame and region). The relative data is normalized (representing the frequency of searches for a given term relative to the volume of search activity) and presented on a scale of 0–100.

For the present study, searches for terms ‘weight’, ‘diet’, ‘fitness’, and ‘smoking’ conducted within the U.S. between January 4^th^, 2004 (first time data made available) through November 28^th^, 2011 (date of data download and analysis) were analyzed. The terms used for this study were chosen as they were both general to the areas of behavior change being explored and were identified as the most commonly searched terms in each area. To identify the most commonly search terms for each area of behavior change, we began by comparing search activity for 5–6 terms commonly found in the literature. For example, before deciding to use the term ‘fitness’, we compared the volume of searches for terms ‘fitness’, ‘fit’, ‘physical activity’, ‘exercise’, ‘gym’ and ‘work out’. Google Insights for Search also provides a list of ‘Top Search Terms’ and ‘Rising Search Terms’ for each entered term. We compared the chosen search terms to the top five search terms and rising search terms provided by Google Insights and then used the most popular term.

### Statistical Analysis

Descriptive statistics (Mean ± S.D.) for the mean relative search frequency per month for each term conducted between 2004 and 2011 was calculated. Using a generalized linear model, we examined whether monthly relative search frequency changed over time (e.g., month of the year). Statistical analyses were performed using SAS 9.3 and significance was set a priori at p<0.05.

## Results

**Figure 1:**
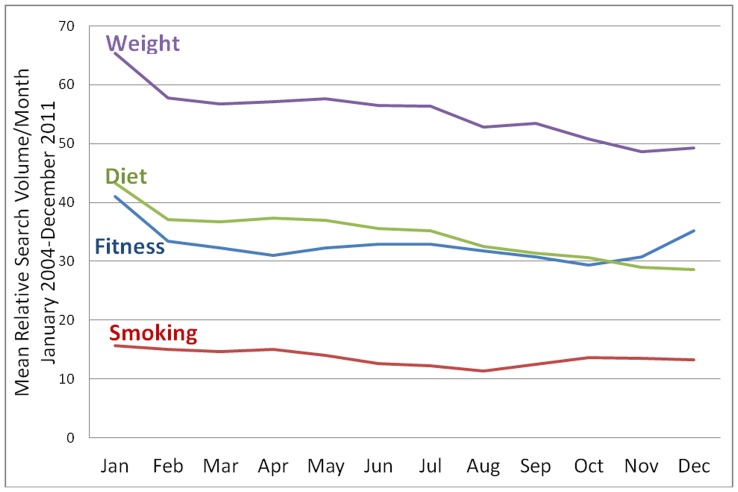
Mean relative search volume per month from 2004 to 2011for terms ‘weight, ‘diet’, ‘fitness’ and ‘smoking’.

As illustrated in Figure 1, seasonality patterns appear to emerge for all four search terms with search activity consistently highest in the month of January (each year). Models of the main effect of month of the year on search activity suggested a significant effect of time on searches for ‘fitness’, ‘diet’ and ‘weight’. Specifically, compared to January, mean searches for all three terms decreased significantly during subsequent months with most significant differences appearing between October (lowest) and January (highest) for ‘fitness’(B = −12.21, SE = 0.95, p<.001), and between December (lowest) and January (highest) for both ‘diet’ (B = −20.17, SE = 1.64, p<.001) and ‘weight’ (B = −17.32, SE = 1.69, p<.001). For ‘smoking’ search activity, significant differences (compared to January) were observed for the months May through December only, with largest differences appearing between August (lowest) and January (highest) (B = −17.42, SE = 3.10, p<0.001).

## Discussion

The findings from this study indicate public interest in four areas of behavior change (i.e., fitness, diet, weight loss, and smoking) fluctuate throughout the year but consistently peak in January. While past studies have explored the use of search query data for detecting the rise of influenza epidemics [6], [10], seasonal trends of depression [7] and changes in smoking habits [8], [9], this study is the first to use search query data to explore trends in diet, weight loss and/or fitness.

The observed increase in smoking searches in January is consistent with the findings of Ayers et al. [8]. Similar increases/trends in search activity during the month of January for terms ‘fitness’ ‘diet’ and ‘weight loss’ were also observed, which is consistent with the timing of the traditional New Year’s resolution [11]. It has been reported that less than half (40%) of those that begin a resolution maintain the behavior change six months later [12]. The rapid declines in search activity for all four search terms immediately following the month of January are supportive of these findings. However, the attribution of monthly search query variations to a New Year’s resolution effect is only speculative and may not illustrate actual motivation for behavior change. Future studies focused on determining whether search query behavior leads to actual behavior change is warranted.

This study also illustrates the potential of using search query data for behavioral medicine and public health purposes. Goel et al. highlighted the value of using search query data suggesting that search queries reveal relevant details about present behaviors and may serve as a tool to predict future behaviors, especially when no other data are available [13]. Because search query data is free, publicly available in nearly real-time and provides future projections of search activity over the coming year, this data could serve researchers, practitioners and policy makers in the fields of public health and behavioral medicine. For example, such data could be used to inform the ideal timing and location of behavioral messages used in public health interventions, media campaigns and/or legislation aimed at improving public health. For instance, Sheffer et al. demonstrated launching a statewide media campaign timed to coincide with temporal smoking-cessation behavioral patterns resulted in increased participation in a statewide tobacco quit line service [13]. Conversely, these data could also be used to identify times and locations of low public interest (i.e., increased need) in a given area which may require additional, targeted and timely messages in order to maintain public interest in these areas across the year. Additionally, Google Insights provides the user with additional contextual information of popular (Top Related Searches) and fast growing (Rising Searches) related terms. Finally, search query information could inform public health researchers and practitioners on specific key words that resonate with individuals in the early stages of readiness to change. For example, when searching for the term ‘exercise’, the top rising search related to this term is ‘P90X’, a popular home fitness program. When searching for the term ‘diet’, the top rising search is ’17 Day Diet’, a recent best-selling book and diet program. These data might suggest that individuals looking to become more active and/or lose weight are highly interested in shorter term programs that they can use in the privacy of their own homes.

The present findings are limited to searches for single terms conducted via Google between the timeframe of January 2004 and November 2011. However, as Google has been the most widely used search engine since 2006 accounting for 66.1% of all searches in October of 2011 [14], we believe these data present a fair representation of the U.S. population’s search activity. These findings are also limited to searches conducted in the U.S. It is likely that differences in search activity for the chosen terms exist at both international and regional levels. Finally, given the process used for identifying the search terms for each area of behavior change, it is possible that the chosen terms may not best represent the targeted areas of behavior change.

Future research in this area should explore more deeply into geographic differences for search activity on these terms. Given the observed seasonality trends for physical activity [15], [16] and known geographic differences in smoking [17] and overweight/obesity [18], geographic differences in search activity likely also exists. A more detailed analysis of search activity would allow for more timely, targeted and potentially more effective behavioral messages to be used in public health interventions and campaigns. Future research should explore the efficacy of messages informed by search query data for improving public health outcomes. Finally, it is recognized that the linkage between search query behavior and actual behavior change is speculative and has yet to be established. In order to validate whether search query data is truly predictive of actual future behavior change, studies that match temporal search query data with temporal health outcomes data are warranted.
